# Association of the Mediterranean Dietary Quality Index with handgrip strength and muscle endurance: A cross‐sectional study

**DOI:** 10.1002/fsn3.2878

**Published:** 2022-04-22

**Authors:** Sanaz Pourreza, Hossein Shahinfar, Elham Bazshahi, Fateme Gholami, Kurosh Djafarian, Sakineh Shab‐Bidar

**Affiliations:** ^1^ Department of Community Nutrition School of Nutritional Sciences and Dietetics Tehran University of Medical Sciences (TUMS) Tehran Iran; ^2^ Department of Nutrition School of Public Health Iran University of Medical Sciences Tehran Iran; ^3^ Department of Clinical Nutrition School of Nutritional Sciences and Dietetics Tehran University of Medical Sciences (TUMS) Tehran Iran

**Keywords:** cross‐sectional studies, mediterranean diet, muscle strength, physical endurance

## Abstract

Mediterranean diet is a healthy eating pattern associated with various health advantages. Different scoring methods of adherence to this diet have been used to investigate the association between the Mediterranean diet and muscle outcomes. The present study aimed to investigate the association of the Mediterranean Dietary Quality Index (Med‐DQI) with handgrip strength (HS) and muscle endurance (ME). The current cross‐sectional study involved 268 Iranian adults aged 18–70 years. Anthropometric measures were evaluated. We used the 168‐item food frequency questionnaire (FFQ) to develop the Med‐DQI score. The Med‐DQI score ranges from 0 to 14 (lower score suggests higher adherence to Mediterranean diet). HS was measured by a digital handgrip dynamometer, and ME was the amount of time to reach a maximum of 50% of grip strength. Multiple regression analysis was used to determine the association of Med‐DQI and its components with HS and ME. Med‐DQI score was not associated with mean handgrip strength (MHS) (*p* = .34) and mean muscle endurance (MME) (*p* = .69) in the crude model. The associations remained insignificant after the adjustment of covariates (*p* = .16, .31, respectively). Among the components of Med‐DQI, cholesterol, meats, fish, and cereals were positively correlated with MHS (*p* < .001 for all). Meats (*p* = .02), olive oil (*p* < .001), cereals (*p* = .04), fruits and vegetables (*p* < .001 for all) were also positively correlated with MME. According to our findings, Med‐DQI score is not associated with MHS and MME in a population of Iranian adults. Further longitudinal studies are required to confirm these findings.

## Graphical abstract

- Mediterranean Dietary Quality Index (Med‐DQI) was not associated with handgrip strength (HS) and muscle endurance (ME).
- Among the various components of Med‐DQI, meats, cholesterol, fish, and cereals were positively correlated with mean handgrip strength (MHS).
- Olive oil, fruits and vegetables, meats, and cereals had a positive correlation with mean muscle endurance (MME).

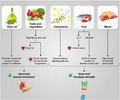

## 1 INTRODUCTION

Muscle mass, strength, and metabolic function are not only important for exercise but are undoubtedly vital for daily activities (Wolfe, 2006). Handgrip strength (HS) is defined as the ability to wield a physical force, while muscle endurance (ME) is the ability of the muscle to keep that level of exertion during a certain period (McArthur et al., 2019; de Zwart et al., 2018). HS starts to decrease from the second and fourth decade of life in men and the fourth decade in women (Cipriani et al., 2012). Poor HS is linked to various chronic diseases such as obesity, insulin resistance and diabetes, and osteoporosis in middle‐aged and older people (Cui et al., 2019; Wolfe, 2006). In obesity, increased triglycerides (TGs), decreased high‐density lipoprotein cholesterol (HDL‐C) with increased small dense low‐density lipoprotein (LDL) are observed due to impaired lipolysis of TG‐rich lipoproteins (Klop et al., 2013). Age, gonadal status, physical activity, and exercise, along with nutrition, play a critical role in muscle health and outcomes (Cipriani et al., 2012; Robinson et al., 2019).

Many observational and intervention studies (Franzke et al., 2018; Granic et al., 2019; Landi et al., 2016; Muir & Montero‐Odasso, 2011) have shown the relationship between nutrition and muscle health by investigating single nutrients (e.g., protein, vitamin D). Since both food and nutrients form a diet, dietary patterns have been increasingly used (Fougère et al., 2016). One of the healthiest dietary patterns is the Mediterranean diet, which has been demonstrated to reduce morbidities of chronic diseases and increase life expectancy (Mariscal‐Arcas et al., 2009). The Mediterranean dietary pattern is defined by a high intake of fruits and vegetables, nuts and legumes, whole grains, fish, olive oil but low intake of saturated fats, reduced intake of dairy products (often in the form of cheese or yoghurt) and meat, and a regular but moderate intake of alcohol, mainly red wine (Di Daniele et al., 2017; Tur et al., 2005). This diet exerts anti‐obesity, antitumor, and anti‐inflammatory benefits via its abundance of fiber, polyunsaturated fats, and bioactive antioxidant compounds including phytochemicals (Di Daniele et al., 2017; Farhangi et al., 2017). The antioxidant and anti‐inflammatory features in conjunction with the direct role of potassium or magnesium in muscle metabolism and physiology would lead to skeletal muscle mass (SMM) preservation as well (Barrea et al., 2019). A Mediterranean Dietary Quality Index (Med‐DQI) created by Gerber (2001), Gerber et al. (2000) emphasizes two separate fat sources (saturated fat and olive oil) and two distinct sources of protein (meat and fish) with reverse scores to estimate diet quality. This index is based on the recommendations and guidelines made by the National Research Council and American Heart Association similar to those prescribed to people at risk of cardiovascular disease (CVD): consumption of 30% or less of total calorie intake from fat, 10% or less from saturated fat, 30 mg/day or less cholesterol, 55% of energy from complex carbohydrates, and 5 servings or more of fruit and vegetables (Gerber et al., 2000). The Med‐DQI has been validated using various nutritional biomarkers, i.e. α‐ and β‐carotenes, vitamin E, eicosapentaenoic acid (EPA), and docosahexaenoic acid (DHA) (Gerber, 2001).

A large number of studies have reported the association of adherence to the Mediterranean diet using various scoring methods with cardiovascular risk factors (Vitale et al., 2018), cancer (de Lorgeril et al., 1998), nonalcoholic fatty liver disease (NAFLD) (Zelber‐Sagi et al., 2017), cognitive health (Aridi et al., 2017), depression (Hernández‐Galiot & Goñi, 2017), muscle strength (Barrea et al., 2019), and physical performance (Fougère et al., 2016). However, no study is available examining the association of Med‐DQI with HS and ME. Since nutrition is of paramount importance in preserving muscle mass, and muscle quality is strongly correlated with all‐cause mortality, disability, and increased duration of hospital stay(Barrea et al., 2019), we aimed to assess the association between Med‐DQI, HS, and ME in a cross‐sectional study of Iranian adults.

## 2 MATERIALS AND METHODS

### 2.1 Study design and participants

The present cross‐sectional study was conducted on 268 adults (117 males and 151 females) aged 18–70 living in Tehran, Iran. To determine the sample size, we have considered the correlation of protein intake with muscle handgrip as outcome variable (Huschtscha et al., 2021). We calculated the sample size based on correlation coefficients ≥0.3 at the 5% significance level and with 80% power. The following formula was used for sample size calculation and the sample size of 84 people was obtained: $N=\frac{{\left(z_{1‐\frac{\alpha }{2}}+z_{1‐\beta \times \sqrt{1‐r^{2}}}\right)}^{2}}{r^{2}}+3$. To compensate for the potential exclusion of participants due to under‐ and overreporting of total energy intake, or attrition due to other reasons, the final sample size of 270 participants was selected for inclusion. Recruitment methods involved announcement, distribution of flyers, and information sessions held at residential facilities from February 2018 to July 2019. Participants were chosen using convenience sampling. Individuals were included if they met the following criteria: (1) 18–70 years and (2) no alcohol or drug addiction. Exclusion criteria consisted of: (1) lactating or pregnant women, (2) adults with chronic diseases such as kidney, liver, and pulmonary disease, diabetes, hormonal and cardiovascular diseases, (3) active infectious or inflammatory diseases, (4) following certain diets (weight control diets), taking any special medications or supplements (supplements with thermogenic ingredients such as caffeine and green tea, supplements affecting weight, hormones, and sedatives), (5) malnutrition, and (6) any disorder affecting the exercise test (including atopy, asthma, myocardial infarction,…), inability to walk, joint problems, and disabilities. Each participant was completely informed about the study protocol. All procedures were approved by the Ethics Committee of the Tehran University of Medical Sciences (Ethic Number: IR.TUMS.VCR.REC.1396.4085). All participants signed a written informed consent before the beginning of the study. A questionnaire for assessing demographic information (age, education, income, and marital status), lifestyle (smoking), medical history, and dietary habits was completed by each participant.

### 2.2 Physical activity

A short form of the International Physical Activity Questionnaire (IPAQ) (Moghaddam et al., 2012) validated before was used to assess the level of physical activity for each participant. Based on calculated metabolic equivalents (METs), the participants were classified into three levels: very low physical activity (<600 MET‐min/week), low physical activity (600–3,000 MET‐min/week), and moderate and high physical activity (>3,000 MET‐min/week) (Wareham et al., 2003).

### 2.3 Dietary intake

Participants’ dietary intakes were assessed by a valid and reliable semiquantitative food frequency questionnaire (FFQ) (Mirmiran et al., 2010), which contained 168 food items. This FFQ consisted of a list of foods with standard serving sizes commonly consumed by the Iranian population. A trained dietitian asked participants about the frequency intake of each item during the previous year on a daily, weekly, or monthly basis. These reports were converted to daily intakes. Portion sizes of consumed foods were converted to grams by using household measures (Ghaffarpour et al., 1999). Mean energy and nutrient intakes of each item were calculated using Nutritionist IV software, modified for Iranian foods (version 7.0; N‐Squared Computing).

### 2.4 Med‐DQI

We calculated the Med‐DQI score based on the method proposed by Gerber (2001), Gerber et al. (2000). This score was obtained from 7 main components: saturated fatty acids, cholesterol, meats, olive oil, fish, cereals, vegetables and fruits. For the meats group, red and processed meat and white meat were included. All kinds of fish and tuna were added to the fish group. Pasta and all types of bread were included in the cereals group. To create Med‐DQI, a score of 0, 1, or 2 was attributed to each food group depending on the amount of daily consumption (Table 1). The final score was reported as a sum of all the nutrient scores between 0 and 14. A lower score shows the better nutritional quality and higher compliance with the Mediterranean diet pattern.

**TABLE 1:** Construction of the score for the Mediterranean Dietary Quality Index

Scores	0	1	2
Saturated fatty acids, % energy	<10	10–13	>13
Cholesterol, mg	<300	300–400	>400
Meats, g	<25	25–125	>125
Olive oil, ml	>15	15–5	<5
Fish, g	>60	60–30	<30
Cereals, g	>300	300–100	<100
Fruits + vegetables, g	>700	700–400	<400

### 2.5 Anthropometric measurements and body composition

A body composition analyzer (InBody 770, Biospace) was used to measure weight, body mass index (BMI), waist circumference (WC), waist to hip ratio (WHR), and body composition components (including fat‐free mass (FFM), fat mass (FM), and percent body fat (PBF)). Participants were required not to exercise vigorously, consume too much food or fluid, and be in a fasting condition with an empty bladder at the time of analysis. Height was measured with a tape when the participants were in a standing position and bare feet while their shoulders were in a normal position (Janssen et al., 2002). A digital handgrip dynamometer (Saehan, model SH5003; Saehan Corporation) was utilized to measure HS. Participants were asked to squeeze the dynamometer as hard as possible to reach the maximum amount of static force while standing with arms by their side and a flexed elbow. The process was repeated three times for each hand. Altogether, measurements of handgrip strength of right hand (HSR) and handgrip strength of left hand (HSL) were used for data analyses (Pedrero‐Chamizo et al., 2013). Subjects were then instructed to sustain the maximal pressure while squeezing the digital handgrip dynamometer. The time (in s) for grip strength to reach 50% of the maximum was recorded as ME (Bautmans & Mets, 2005).

### 2.6 Statistical analysis

Participants were categorized by tertiles of Med‐DQI. To investigate the differences among the tertiles, we used a one‐way analysis of variance (anova) for continuous variables and chi‐square test for categorical variables. We used the analysis of covariance (ancova) for comparing dietary intake across the tertiles of Med‐DQI with adjustment for age, sex, and energy intake, and also for comparing HS and ME among the tertiles of Med‐DQI with adjustment for known potential confounders including age, sex, BMI, smoking, physical activity, and total energy intake. For evaluating the correlation of Med‐DQI components and body composition parameters with mean handgrip strength (MHS) and mean muscle endurance (MME), Pearson's correlation was used. Multiple regression analysis was also applied to determine the association of Med‐DQI and its components with HS and ME. Potential confounding variables from the literature or those with sufficient mechanistic reason including age, sex, BMI, smoking (smoker/nonsmoker), physical activity (low/moderate/high), energy, and protein intake were controlled in the adjusted model. Statistical Package for the Social Sciences (SPSS version 25; SPSS Inc.) was used to perform statistical analyses. A value of *p* < .05 was considered to be statistically significant.

## 3 RESULTS

The total number of 268 participants was divided according to Med‐DQI tertiles. Mean of Med‐DQI, MHS, and MME was 6.41, 31.1, and 124, respectively. General characteristics of individuals are presented in Table 2. Mean age of participants was 36.5 ± 13.1 years, and 56.3% were women. Age (*p* < .001) and height (*p* = .02) were significantly different among the tertiles. Compared with those in the first tertile, the number of participants in the highest tertile of Med‐DQI was more likely to be married (*p* = .01). There were no statistically significant differences in the mean of other variables during the Med‐DQI tertiles. Although not significant, participants in the highest tertiles of Med‐DQI had higher FFM (51.7 ± 12.0, *p *= .35) and weight (73.5 ± 17.8, *p* = .83) compared to individuals in the lowest tertiles (48.6 ± 11.7 and 71.9 ± 14.2, respectively).

**TABLE 2:** General characteristics of participants by tertiles (T) of Med‐DQI

Range	All = 268	Tertiles of Med‐DQI			*p*‐value*
		T1 (*n* = 77) (2–5)	T2 (*n* = 126) (6–7)	T3 (*n* = 65) (8–13)	
	Mean ± SD	Mean ± SD	Mean ± SD	Mean ± SD	
Height (cm)	168 ± 9.99	165 ± 10.7	168 ± 9.87	169 ± 8.80	.**02**
Age (years)	36.5 ± 13.1	44.3 ± 14.9	33.8 ± 10.7	32.8 ± 11.3	**<.001**
FFM (kg)	50.1 ± 12.6	48.6 ± 11.7	50.2 ± 13.5	51.7 ± 12.0	.35
FM (kg)	22.4 ± 9.4	23.2 ± 9.4	22.3 ± 8.7	21.7 ± 10.6	.65
Weight (kg)	72.7 ± 16.1	71.9 ± 14.2	72.8 ± 16.3	73.5 ± 17.8	.83
WC (cm)	89.6 ± 12.6	89.7 ± 11.3	89.4 ± 12.0	89.8 ± 15.0	.97
WHR	0.90 ± 0.06	0.90 ± 0.06	0.90 ± 0.05	0.90 ± 0.07	.92
BMI (kg/m^2^)	25.6 ± 4.68	26.1 ± 4.45	25.4 ± 4.58	25.2 ± 5.15	.49
Sex (%)					
Male	43.7	39.0	41.3	53.8	.15
Female	56.3	61.0	58.7	46.2	
Marital status (%)					
Single	43.1	29.9	45.6	53.8	.**01**
Married	56.9	70.1	54.4	46.2	
Smoking (%)					
Nonsmoker	86.6	90.9	88.1	78.5	.07
Former and current smokers	13.4	9.1	11.9	21.5	
Physical activity (%)					
Low	38.1	41.6	38.1	33.8	.83
Medium	41.4	39.0	42.9	41.5	
High	20.5	19.5	19.0	24.6	
Diabetes (%)					
Yes	3.4	6.5	2.4	1.5	.18
No	96.6	93.5	97.6	98.5	
CVD (%)					
Yes	2.2	5.3	1.6	0.0	.08
No	97.8	94.7	98.4	100	

Note

Values are based on average ± SD or reported percentage

The bold values are the ones under *p* < .05 considered as statistically significant.

Abbreviations: Med‐DQI, Mediterranean Dietary Quality Index; SD, standard deviation; FFM, fat‐free mass; FM, fat mass; WC, waist circumference; WHR, waist to hip ratio; BMI, body mass index; CVD, cardiovascular disease.

* One‐way anova for quantitative data and chi‐squared test for qualitative data have been used.

In Table 3, dietary intakes of the participants according to the tertiles of Med‐DQI are reported. There was no significant difference in energy intake across the tertiles of Med‐DQI (*p* = .34). Dietary intakes of carbohydrates (*p* < .001), total fat (*p* < .001), thiamin (*p* < .001), riboflavin (*p* = .04), niacin (*p* = .04), folate (*p* = .01), vitamin C (*p* < .001), and calcium (*p* = .02) were significantly different across the tertiles. Although not significant, participants at the highest tertile of Med‐DQI had greater means of vitamin D, vitamin E, and Zn intakes.

**TABLE 3:** Total energy and nutrients intake according to the tertiles (T) of Med‐DQI

Range	All = 268	Tertiles of Med‐DQI			*p*‐value*
		T1 (*n* = 77) (2–5)	T2 (*n* = 126) (6–7)	T3 (*n* = 65) (8–13)	
	Mean ± SD	Mean ± SD	Mean ± SD	Mean ± SD	
Energy (kcal/d)	2,391 ± 965	2,531 ± 1,114	2,313 ± 904	2,376 ± 881	.34
Carbohydrates (% energy)	57 ± 8.04	61.8 ± 7.26	57.3 ± 7.06	50.7 ± 6.42	**<.001**
Protein (% energy)	15.2 ± 3.52	14.9±	14.9±	16.3±	.07
Total fat (% energy)	29.8 ± 7.51	25.7 ± 6.09	29.8 ± 6.87	34.8 ± 7.30	**<.001**
Thiamin (mg)	1.88 ± 0.95	2.17 ± 1.25	1.83 ± 0.81	1.63 ± 0.68	**<.001**
Riboflavin (mg)	1.76 ± 1.00	1.79 ± 1.25	1.65 ± 0.86	1.94 ± 0.88	.**04**
Niacin (mg)	22.7 ± 12.8	25.1 ± 18.9	21.9 ± 9.65	21.4 ± 8.66	.**04**
Vitamin B6 (mg)	1.49 ± 0.86	1.70 ± 1.14	1.40 ± 0.69	1.44 ± 0.71	.20
Folate (µg)	318 ± 178	374 ± 224	296 ± 154	295 ± 146	.**01**
Vitamin D (µg)	2.35 ± 2.56	2.47 ± 3.23	2.17 ± 2.19	2.57 ± 2.33	.60
Vitamin E (mg)	4.45 ± 3.17	4.58 ± 2.98	4.25 ± 2.30	4.69 ± 4.59	.59
Vitamin A (µg)	1,380 ± 1,036	1,630 ± 1,014	1,216 ± 812	1,401 ± 1,356	.22
Vitamin C (mg)	138 ± 78.2	187 ± 88.5	121 ± 63.0	115 ± 66.3	**<.001**
Zn (mg)	9.84 ± 5.37	10.0 ± 7.01	9.28 ± 4.61	10.7 ± 4.36	.06
Se (µg)	0.04 ± 0.03	0.04 ± 0.03	0.04 ± 0.03	0.04 ± 0.03	.22
Fe (mg)	21.7 ± 11.1	23.4 ± 11.0	21.5 ± 12.3	20.0 ± 8.36	.19
Ca (mg)	1,034 ± 546	1,106 ± 539	942 ± 485	1,128 ± 640	.**02**
Magnesium (mg)	288 ± 123	320 ± 141	273 ± 110	281 ± 119	.12

Abbreviations: Med‐DQI, Mediterranean Dietary Quality Index; SD, standard deviation.

The bold values are the ones under *p* < .05 considered as statistically significant.

* P obtained from analysis of covariance (ANCOVA) test adjusted by age, sex, and energy intake. Data for energy intake have just been adjusted for age and sex.

Table 4 presents the multivariate‐adjusted means for HS and ME across the tertiles of Med‐DQI. Before and after controlling various covariates including age, sex, BMI, smoking, physical activity, protein and energy intake, we found no difference. However, in the highest tertile of Med‐DQI, participants had higher MHS (31.9 ± 12.0, *p* = .34) and HSR (33.5 ± 12.5, *p* = .36) compared to the lowest tertile (29.4 ± 11.5 and 30.7 ± 12.3, respectively).

**TABLE 4:** The multivariate‐adjusted means for HS and ME across tertiles (T) of Med‐DQI

Range	All = 268	Tertiles of Med‐DQI			*p*‐value*	*p*‐value**	*p*‐value***
		T1 (*n* = 77) (2–5)	T2 (*n* = 126) (6–7)	T3 (*n* = 65) (8–13)			
	Mean ± SD	Mean ± SD	Mean ± SD	Mean ± SD			
MHS (kg)	31.1 ± 11.7	29.4 ± 11.5	31.6 ± 11.6	31.9 ± 12.0	0.34	0.20	0.16
HSR (kg)	32.3 ± 12.2	30.7 ± 12.3	32.7 ± 12.0	33.5 ± 12.5	0.36	0.18	0.32
HSL (kg)	29.8 ± 11.5	28.1 ± 11.1	30.5 ± 11.6	30.4 ± 11.7	0.32	0.23	0.09
MME (s)	124 ± 67.2	129 ± 46.1	122 ± 82.1	120 ± 53.2	0.69	0.42	0.31
MER (s)	128 ± 53.6	134 ± 48.4	125 ± 55.2	128 ± 56.6	0.53	0.55	0.43
MEL (s)	119 ± 99.4	125 ± 50.9	120 ± 132	111 ± 55.7	0.75	0.45	0.32

Abbreviations: HS, handgrip strength; ME, muscle endurance; Med‐DQI, Mediterranean Dietary Quality Index; SD, standard deviation; MHS, mean handgrip strength; HSR, handgrip strength of right hand; HSL, handgrip strength of left hand; MME, mean muscle endurance; MER, muscle endurance of right hand; MEL, muscle endurance of left hand.

* Crude model

** *p* for trend

*** Obtained from analysis of covariance (ancova) test adjusted by age, sex, body mass index (BMI), smoking, physical activity, protein and energy intake.

The correlation between the Med‐DQI and its components with MHS and MME is shown in Table 5. The correlation between cholesterol (*r* = .32), meats (*r* = .22), fish (*r* = .21), and cereals (*r* = .26) with HS was statistically significant (*p* < .001 for all). Among the body composition parameters, FFM had a strong correlation (*r* = .8, *p* < .001) with MHS, while the correlation of FM (*r* = −.16, *p* = .009), PBF (*r* = −.56, *p* < .001), and WHR (*r* = .2, *p* = .001) was weak. There was no significant correlation between body composition components and MME. Meats (*r* = .14, *p* = .02), olive oil (*r *= .23, *p* < .001), cereals (*r* = .13, *p* = .04), vegetables and fruits (*r* = .24, *p* < .001) had a weak significant correlation with MME.

**TABLE 5:** Correlation of MHS and MME with Med‐DQI, its components and several parameters of body composition

	MHS		MME	
	*r* ^a^	*p*‐value	*r* ^a^	*p*‐value
Med‐DQI	.07	.22	−.10	.08
Saturated fatty acid (%)	−.03	.57	−.07	.26
Cholesterol (mg)	.32	**<.001**	.04	.50
Meats (g)	.22	**<.001**	.14	.**02**
Olive oil (ml)	.01	.86	.23	**<.001**
Fish (g)	.21	**<.001**	.07	.22
Cereals (g)	.26	**<.001**	.13	.**04**
Vegetables + fruits (g)	.07	.24	.24	**<.001**
FFM (kg)	.80	**<.001**	.06	.31
FM (kg)	−.16	.**009**	.07	.23
LBM (kg)	−.10	.09	−.07	.27
PBF (%)	−.56	**<.001**	.04	.52
WHR	.20	.**001**	−.01	.85

Abbreviations: MHS, mean handgrip strength; MME, mean muscle endurance; Med‐DQI, Mediterranean Dietary Quality Index; FFM, fat‐free mass; FM, fat mass; LBM, lean body mass; PBF, percent body fat; WHR, waist to hip ratio. 1

^a^ Data are Pearson correlation coefficients.

Table 6 reports the association between Med‐DQI components and Med‐DQI with MHS and MME in multiple regression analysis models. For MHS, cholesterol in both models showed a positive significant association (*β* ± SE = 0.01 ± 0.002, *p* < .001 for model l, *β* ± SE = 0.006 ± 0.002, *p* = .008 for model 2). However, meats, fish, and cereals showed a significant relationship only in model 1 (*p* < .001 for all). For MME there was a significant positive correlation in both models for olive oil (*β* ± SE = 5.64 ± 1.48, *p* < .001 for model l, *β* ± SE = 4.24 ± 1.52, *p* = .006 for model 2). But meats (*β* ± SE = 0.24 ± 0.10, *p* = .02), cereals (*β* ± SE = 0.03 ± 0.01, *p* = .04), fruits and vegetables (*β* ± SE = 0.04 ± 0.01, *p* < .001) showed a significantly positive relationship only in model 1.

**TABLE 6:** Multiple regression analysis models investigating the association of Med‐DQI components and Med‐DQI with MHS and MME

	MHS					MME				
	*β* ^a^	SE	*p*‐value	*t*	95% CI	*β* ^a^	SE	*p*‐value	*t*	95% CI
Saturated fatty acids (%)										
Model 1^b^	−0.13	0.24	.57	−0.56	−0.62 to 0.34	−1.63	1.46	.26	−1.11	−4.52 to 1.25
Model 2^c^	−0.10	0.16	.53	−0.62	−0.41 to 0.21	0.45	1.50	.76	0.30	−2.50 to 3.40
Cholesterol (mg)										
Model 1	0.01	0.002	**<.001**	5.65	0.009 to 0.01	0.01	0.01	.50	0.67	−0.02 to 0.04
Model 2	0.006	0.002	.**008**	2.67	0.001 to 0.01	0.008	0.01	.69	0.39	−0.03 to 0.04
Meats (g)										
Model 1	0.06	0.01	**<.001**	3.69	0.03 to 0.10	0.24	0.10	.**02**	2.26	0.03 to 0.46
Model 2	0.006	0.01	.63	0.47	−0.01 to 0.03	0.23	0.11	.05	1.96	0.00 to 0.46
Olive oil (ml)										
Model 1	0.04	0.26	.86	0.16	−0.47 to 0.56	5.64	1.48	**<.001**	3.78	2.70 to 8.57
Model 2	0.09	0.17	.57	0.56	−0.23 to 0.43	4.24	1.52	.**006**	2.79	1.24 to 7.23
Fish (g)										
Model 1	0.14	0.04	**<.001**	3.61	0.05 to 0.15	0.29	0.23	.22	1.22	−0.17 to 0.76
Model 2	0.01	0.02	.66	0.43	−0.04 to 0.06	0.24	0.24	.31	1.00	−0.23 to 0.72
Cereals (g)										
Model 1	0.01	0.002	**<.001**	4.84	0.007 to 0.01	0.03	0.01	.**04**	2.60	0.002 to 0.06
Model 2	0.003	0.002	.24	1.17	−0.002 to 0.007	0.006	0.02	.75	0.30	−0.03 to 0.04
Vegetables + fruits (g)										
Model 1	0.002	0.002	.24	1.16	−0.002 to 0.006	0.04	0.01	**<.001**	3.96	0.02 to 0.06
Model 2	0.002	0.001	.26	1.12	−0.001 to 0.005	0.02	0.01	.13	1.52	−0.006 to 0.04
Med‐DQI										
Model 1	0.52	0.43	.22	1.21	−0.32 to 1.38	−4.40	2.57	.08	−1.70	−9.47 to 0.67
Model 2^d^	−0.30	0.29	.29	−1.04	−0.88 to 0.27	0.19	2.74	.94	0.07	−5.21 to 5.61

Abbreviations: Med‐DQI, Mediterranean Dietary Quality Index; MHS, mean handgrip strength; MME, mean muscle endurance; SE, standard error; CI, confidence interval.

The bold values are the ones under *p* < .05 considered as statistically significant.

^a^ β coefficient obtained from linear regression.

^b^ Crude.

^c^ Adjusted for age, sex, body mass index, smoking, physical activity, and energy intake.

^d^ Additionally adjusted for protein intake.

## 4 DISCUSSION

This cross‐sectional study assessed the association of Med‐DQI with HS and ME. According to the components of Med‐DQI, consumption of cholesterol, meats, fish, and cereals was positively associated with MHS. MME also had a significant positive association with the intake of meats, olive oil, cereals, fruits and vegetables. However, we failed to find a significant association between Med‐DQI, HS, and right‐ and left‐hand endurance. To the best of our knowledge, this is the first study to investigate the association of Med‐DQI with muscular fitness. Previously, four studies have been published investigating the association of the Med‐DQI and other outcomes such as the inflammatory potential of diet (Abbasalizad Farhangi & Najafi, 2018), metabolic risk factors for CVD (Farhangi et al., 2017), vascular endothelial growth factor (VEGF) +405 G/C gene polymorphism (Hajiluian et al., 2017), and the blood levels of homocysteine, folate, and vitamin B12 (Visekruna et al., 2015).

A growing body of literature is assessing the association of different dietary patterns, HS, and ME. Among various types of dietary patterns, the Mediterranean diet has been reported to have beneficial effects on the risk of CVD, cancer, and other chronic diseases (Carlos et al., 2018; Davis et al., 2015). These effects are due to its high amounts of fish, olive oil, whole grains, fruits and vegetables, nuts, and legumes (Trichopoulou & Lagiou, 1997; Widmer et al., 2015). Med‐DQI has been validated by the use of nutritional biomarkers, which makes it an appropriate tool for measuring dietary quality (Gerber, 2001). This index considers all the major features of the Mediterranean diet based on the recommendations of the National Research Council and American Heart Association (Gerber et al., 2000; Satalic et al., 2004).

In the current study, we found no significant association between Med‐DQI, HS, and ME. In line with our findings, Kelaiditi et al. (2016) reported no statistically significant association between the Mediterranean diet score and handgrip strength in a large‐scale cross‐sectional study conducted among women aged 18–79 years. Fougère et al. (2016) could not find a correlation between Mediterranean diet score and handgrip strength in a group of 77 years and older men and women, either. In another study, Talegawkar et al. (2012) found that higher adherence to the Mediterranean‐style diet is associated with some components of frailty such as lower physical activity and walking speed; however, these associations were not detected for the feeling of exhaustion and HS in Italian people aged 65 years or older. Bollwein et al. (2013) drew the same conclusion in a German population of 75 years and older.

In contrast to our findings, in a cross‐sectional observational study of 84 elderly women, Barrea et al. (2019) reported a positive association between adherence to the Mediterranean diet and HS. This association was still significant after adjusting for BMI in the participants with high and low adherence to the Mediterranean diet. Zbeida et al. (2014) also found a significant relationship between higher adherence to the Mediterranean diet and better HS in a data of American population aged 60 years and older from US National Health and Nutrition Survey (NHANES). In a study by Rahi et al. (2018), after 2 years of follow‐up, older adults, who were more adherent to the Mediterranean diet, had a reduced risk of poor HS, slowness, and low physical activity.

Although Med‐DQI as a whole was not associated with MHS and MME, several components of this score were significantly correlated with MHS and MME. We found that MHS was correlated with the intake of cholesterol, meats, fish, and cereals, while MME had a positive correlation with meats, olive oil, cereals, fruits and vegetables consumption. Increasing evidence reveals that oxidative stress and inflammation are the links between the Mediterranean diet and muscle outcomes (Cesari et al., 2004; Fanò et al., 2001; Jenny et al., 2012; Kelaiditi et al., 2016). Catabolism of muscle fibers gets quicker in the presence of inflammation; thus, muscle fibers and physical performance weaken over time (Ferrucci et al., 1999). With its great content of antioxidant micronutrient and polyphenolic compounds, the Mediterranean diet poses a striking influence on HS and ME (Talegawkar et al., 2012). Regarding Med‐DQI components, since cholesterol is vital for the formation of lipid rafts, higher dietary cholesterol can improve cellular signaling pathways and result in muscle hypertrophy (Riechman et al., 2007), which may in turn boost HS. Higher amounts of fish consumption are related to increased HS (Robinson et al., 2008). Not only is fatty fish a major source of omega‐3 fatty acids, but it is also considered to be rich in dietary vitamin D (Robinson et al., 2008). Polyunsaturated fatty acid (PUFA) from fatty fish decreased inflammatory markers such as interleukin 6 (IL‐6), interleukin 1β (IL‐1β), and tumor necrosis factor alpha (TNF‐α), as reported in an interventional study by Meydani et al. (1993). Besides, calcitriol, the activated form of vitamin D, can bind to the nuclear receptors of skeletal muscle cells, boost protein synthesis, and lead to muscle cell growth (Wilhelm‐Leen et al., 2010).

Beasley et al. (2010), in a cohort study of 24,417 women aged between 65 and 79, detected that higher protein consumption is strongly associated with frailty. Moreover, Daly et al. (2014) reported that 4 months of progressive resistance training combined with a diet rich in lean red meat could significantly promote HS. Protein intake equal to or more than 1 g/kg BW was associated with muscle weakness, slowness, and low physical activity in older adults aged 65 and over (Rahi et al., 2016). A possible mechanism that can be suggested for the impact of meat intake on the preservation of muscle and lean body mass is that meats contain a high amount of essential amino acids, which are prime elements of muscle synthesis (Volpi et al., 2003; Wolfe et al., 2008). Nevertheless, Formica et al. (2020), in a recent randomized controlled trial (RCT), found that 24 weeks of resistance‐based exercise training program along with the intake of lean red meat did not affect HS and function. This could be explained due to the higher baseline intake of red meat (1.25 g/kg) in Formica's study in comparison to the baseline intake of Daly's study (1.1 g/kg) (Formica et al., 2020).

Another key component of the Mediterranean diet is olive oil, rich in phenolic compounds, particularly hydroxytyrosol, tocopherol, or carotenoids (Esquius et al., 2019). The anti‐inflammatory and antioxidant features of these compounds in conjunction with hydroxytyrosol that intensifies the expression of all mitochondrial respiratory chain complexes, and induces mitochondrial biogenesis pathway, lead to an enhancement of central control of ventilation and, thus, a progression of endurance performance (Esquius et al., 2019; Hao et al., 2010).

Fruits and vegetables are other advantageous parts of a Mediterranean‐style diet. Fruits and vegetables are the main sources of antioxidants such as carotenoids and vitamin C in the Mediterranean diet (Bollwein et al., 2013). Many studies have investigated the association between these micronutrients and physical performance. Saito et al. (2012), in a cross‐sectional study of 70 and older Japanese women, revealed a positive correlation between higher plasma vitamin C and HS and physical performance. In addition, higher serum carotenoids as a marker of higher consumption of fruits and vegetables were reported to have a relationship with higher walking speed (Alipanah et al., 2009). Apart from the antioxidant and anti‐inflammatory role of these nutrients, there might be other potential mechanisms for the connection of fruits and vegetables with better endurance. First, their high content of fiber and low amount of fat result in the reduction of body fat and an increase in aerobic capacity. Second, they can decrease blood viscosity and ameliorate blood flow and oxygenation of the body tissues. As a result, these factors could lead to the development of endurance (Barnard et al., 2019).

Another important finding of the present study is the association between HS and composition parameters including FM, FFM, PBF, and WHR. In line with our finding, Cossio‐Bolanos et al. (2020) reported a negative association between FM and relative grip strength among 1,685 children and adolescents of Chile. Sartorio et al. (2002) also detected a statistically significant correlation between handgrip strength and FFM in children of both genders aged 5–15 years. In a cross‐sectional study of elderly women, Nonaka et al. (2018) reported that grip strength is negatively influenced by FM. The hypotheses behind these results are that cytokine secretion such as TNF‐α from adipose tissue catabolizes muscle fibers, which results in a decline of muscle mass (Koster et al., 2011; Nonaka et al., 2018). Furthermore, with the increase of fat mass, insulin resistance occurs, resulting in a procatabolic impact on muscle (Roubenoff & Hughes, 2000).

An important point that should be kept in mind is that age and gender are determining factors in cross‐sectional studies (Barrea et al., 2019; Kelaiditi et al., 2016), and they might be possible reasons for inconsistent results. The majority of studies about the Mediterranean diet and HS (Alipanah et al., 2009; Barrea et al., 2019; Beasley et al., 2010; Daly et al., 2014; Saito et al., 2012) were conducted among elderly women. Women are more likely to choose healthy foods e.g., fruits and vegetables (Westenhoefer, 2005). Besides, as people grow older, they care more about health and natural content (Schliemann et al., 2019). However, our population consists of both genders with a mean age of 36.5 years. Different study designs, baseline physical activity, race, and habitual intake of nutrients are other reasons for discrepancies in the results.

The major strength of this study is its novelty since this is the first study to evaluate the association between Med‐DQI score, HS, and ME. In addition, the standard protocols were used to assess HS and ME, dietary intakes, and physical activity with no risk of bias. On the other hand, some limitations should be acknowledged. First, the cross‐sectional nature of the study prevents us from making cause‐and‐effect relationships. Second, the small sample size is a restriction for generalizing the results. Third, having a wide age range is another limitation since age is a determining factor for handgrip strength and endurance. Lastly, we could not control residual confounders, although we controlled major confounding variables that could affect the associations.

In conclusion, the results from this study indicate that greater adherence to Med‐DQI‐style diet was not associated with HS and ME. However, several components of Med‐DQI, including cholesterol, meats, fish, and cereals, were positively correlated with MHS, and MME had a positive correlation with meats, olive oil, cereals, fruits and vegetables. Further longitudinal studies are required to confirm these findings.

## 5 ETHICAL REVIEW

The study protocol was approved by the ethical standards of the Tehran University of Medical Sciences (Ethic Number: IR.TUMS.VCR.REC.1396.4085), which approved the protocol and informed consent form. Written informed consent was obtained from all study participants.

## CONFLICT OF INTEREST

None of the authors had a conflict of interest.

## AUTHOR CONTRIBUTIONS

**Sanaz Pourreza:** Methodology, Statistical analysis, Manuscript drafting; **Hossein Shahinfar:** Conceptualization, Methodology, Manuscript drafting; **Elham Bazshahi:** Manuscript drafting; **FatemeGholami:** Manuscript drafting; **KuroshDjafarian:** Supervision, **Sakineh Shab‐Bidar:** Supervision, Review, and Editing.

## Data Availability

The data generated or analyzed during the current study are not publicly available but are available from the corresponding author upon reasonable request.

## Footnotes

1 The bold values are the ones under *p* < .05 considered as statistically significant.
